# How to Extract a Diseased and Lost Eye

**Published:** 1872-01

**Authors:** Julian J. Chisolm

**Affiliations:** Baltimore, Maryland, Professor of Eye and Ear Surgery in the University of Maryland; Surgeon in Charge of Baltimore Eye and Ear Institute; Ophthalmic Surgeon to Baltimore Infirmary, etc.


ATLANTA

### MEDICAL AND SURGICAL JOURNAL.

NEW SERIES.

VOL. IX.—JANUARY, 1872.—NO. 10.

### ORIGINAL COMMUNICATIONS.

### HOW TO EXTIRPATE A DISEASED AND LOST EYE.

BY JULIAN J. CHISOLM, M.D., BALTIMORE, MARYLAND,
Professor of Eye and Ear Surgery in the University of Maryland; Surgeon in Charge of Baltimore Eye and Ear Institute; Ophthalmic Surgeon to Baltimore Infirmary, etc.

The operation for the removal of an injured and painfully-diseased eye, in which there is no sight, is now very frequently undertaken, and the results are in the highest degree satisfactory when the operation has been skillfully performed in accordance with the method laid down by ophthalmic surgeons. When these steps are carefully followed, the patient recuperates so rapidly that he is not confined to his room a single day, and at the end of a week can be dismissed from further treatment, having a socket lined by a normal mucous membrane, and in the best condition to receive an artificial eye. As this is the end longed for by those who undergo this ordeal, a few simple rules, not found in books of general surgery, will enable any practitioner to enucleate a lost and painful eye with credit to himself and benefit to the patient.

As ordinarily performed—a kind of gouging operation—the proceeding is one exciting terror in the patient, and is very unsatisfactory to the operator; the very thought of it excites a shudder of apprehension and a well-grounded feeling of horror. This bad but still generally-adopted method consists in separating the lids by the aid of an assistant, and in splitting the outer angle of the lids, that these may be drawn out of the way and protected against injury. Then the sharp points of a curved scissors are entered in the deep grooves between the lids and the ball of the eye. They cut their way until the blades are buried deeply in the socket, regardless of what they are cutting, provided the bony walls themselves are not broken through. Finally a mass of flesh is separated and extracted, consisting of the eye-ball surrounded by its muscular and fat connections, leaving behind a deep chasm, which must fill up by the slow and painful process of granulation. For a long time there remains a pus-discharging socket, a great annoyance to the patient. In time this cavity becomes dry; the lids are sunken, with their inner surface more or less adherent to the socket surface, with the space so encroached upon by adhering bands that no room remains for the comfortable wearing of an artificial eye, even had the upper lid retained some motion through the accidental escape of the levator palpebræ.

For some years past ophthalmic surgeons have condemned and discarded this careless and dangerous method of enucleation; yet our best text-books in general surgery still adhere to it as the regular method of operative procedure. If we turn to page 771 of "Gaut's Science and Practice of Surgery," an English work of rare excellence, just published in London, (October, 1871), and which is said to embody everything that is good from "Holmes' New Encyclopœdia of Surgery," Erichsen's last edition, as well as from the latest writings of surgeons of greatest repute of our day, we will find the operation for enucleation of the eye-ball described as follows:

"The patient reclining and under the influence of chloroform, an incision is made from the outer commissure of the lids backwards, to a sufficient extent just to give free access to the globe; the lids are then held well apart by retractors or curved spatula in the hands of an assistant. By passing the knife from the incision to the inner commissure above and below the globe, carefully avoiding any exposed portion of either lid, the conjunctiva is entirely divided with the ocular attachment of the levator palpebræ and other surrounding muscles. Then seizing the globe with hooked forceps, and drawing it inwards, the knife enters the orbit externally; the eye is detached, the optic nerve and artery are divided at the apex of the orbit, and the attachment severed internally, thus completing the extirpation of the organ. The lachrymal gland should be removed also; it is commonly involved in the disease of the ball, and is always useless, or its presence would occasion inconvenience afterwards, as I have noticed, by a continued secretion of tears overflowing the cheek. Hemorrhage must be arrested by sluicing with cold water and the application of a graduated compress well-placed upon the artery at the apex of the orbit. The eye-lids are closed and a light bandage drawn around the head, covering the other eye. Opiates will have to be administered from time to time, to relieve the severe penetrating and clasping pain in the head. The compress may be removed in about two days, according to the probability of hemorrhage, and not reapplied unless it should recur. The orbit is to be gently syringed, the wound cleansed, and water dressing applied, or other dressing on ordinary principles of treatment."

If eyes were removed only for malignant disease in which the bulb of the eye-ball is much increased, and where it is necessary to cut widely in order to remove, if possible, all malignant structures which may have infiltrated beyond the eye proper into the socket tissues, then the description of the operation as given by Mr. Gaut may be followed as a good method. But from the present standpoint of ophthalmic surgery, by far the more frequently, the lost eye is removed on account of excessive corneal bulging (staphyloma) or general painful disorganization, or from the fear of or actual presence of sympathetic irritation in the remaining good eye, threatening to destroy its usefulness amidst much suffering. These causes for the removal of the useless and painful eye-ball will outnumber cases of malignant disease, necessitating extirpation, twenty to one. The general operation as detailed by Mr. Gaut, if applied to these most numerous cases, should be considered as bad in its conception, dangerous in its performance, serious in its character, and well-calculated, by its painful sequelæ, to create terror in the minds of those who are suffering from eye trouble, and will often deter them from taking advantage of what ought to be one of the simplest, least painful and most successful operations upon the living body.

In criticising this operation for the removal of the eye as described by Mr. Gaut, we would commence, in the first place, by condemning the use of the knife as a most dangerous instrument when buried in the depths of the orbit. In the second place, experience has shown that the incision at the outer angle is altogether uncalled for, and therefore a needless complication. Thirdly, when the knife is sunk deeply in the conjunctival cul-de-sac, cutting the levator palpebræ in its progress from the outer to the inner angle of the eye, it establishes a permanent drooping of the upper lid, which excludes, ever afterwards, the wearing of an artificial eye. Moreover, the cutting away of nearly all of the conjunctiva leaves no covering for the floor of the socket. Time alone can prepare a covering for the open granulating sore, which must long continue to discharge and give pain. In fact, Mr. Gaut calls special attention to the necessity of giving opium freely, from time to time, "to relieve the severe penetrating and clasping pain in the head." The removal of the lachrymal gland is never called for in simple enucleation, and its presence never gives trouble, as every-day experience amply testifies.

Had Mr. Gaut visited any one of the ophthalmic institutions in London, prior to writing his article on enucleation, and had he described the operation as he would have seen it performed by any one of the many conspicuous ophthalmic surgeons of the metropolis, he would have laid down very different rules for the guidance of practitioners. Moreover, he would have warned surgeons from continuing to follow the old operation as described by himself, which has been so universally condemned by all ophthalmic surgeons, and altogether discarded from their every-day practice.

When properly performed, the removal of an eye-ball becomes one of the simplest operations in surgery, not deserving the title to capital operation. Under chloroform, it is painless, speedy, and nearly bloodless. During its performance, the lids should in no way be implicated in the incisions. The various muscles should be cut away from their eye-ball attachments, and not from their socket connections. Instead of removing them, they are especially retained to aid in the movements of the artificial eye. The levator palpebræ muscle should never be exposed nor molested. The entire conjunctiva should be carefully saved, only the corneal covering being, from necessity, sacrificed; therefore, when the eye-ball, deprived of all of its connections, is lifted out of its bed, the conjunctiva, which was formerly reflected over the globe, falls back upon the raw connective tissue surface, and is found ample to cover it—the opening in this elastic membrane, through which the eye was squeezed, closing by puckering, as would be effected by the drawing of a purse-string. The conjunctiva becomes at once adherent to that entire surface, and so protects the wound by its epithelial coating that the socket appears to heal up, as it were, in a few hours, leaving no raw surface for granulations to spring up on, with the consequent purulent discharges. As the ophthalmic artery is not divided at the apex of the orbit, where it is of largest size, and capable of causing annoying hemorrhage, but in the immediate vicinity of the eye-ball, where only a few of its comparatively small branches are exposed to division, the hemorrhage must be very trivial. The tightly-drawn pressure bandage, which is used to protect the patient against hemorrhage, and which tortures the patient for two days when the surgeon is dreading a flow from the large artery cut at the apex of the socket, is removed in an hour, or even less; and, with its removal, all pain at once ceases, not to return.

After the nauseating effects of chloroform have passed off, so little inconvenience is experienced by the patient, as after-results of this operation, that he may, if required, return to his distant home, or even attend to business, the day after the enucleation. The cases are usually kept under observation for eight or ten days. In no case of simple enucleation have I had occasion to administer a single dose of opium after the operation, and my experience in this operation is now quite extensive, having performed as many as three enucleations in one day.

The proper method for removing a lost and painful eye is as follows:

The patient being fully under the influence of chloroform, the operator introduces under the edge of the lids a wire spring speculum, which expands widely the palpebral opening, folding back the lids under the orbital ridge, and protecting them perfectly from injury, whilst at the same time it gives the most ample space for surgical manipulation. The surgeon, standing behind the head of the patient, with a fine-toothed forceps in the left hand, seizes and draws forward a fold of conjunctiva near the corneal periphery. With a pair of curved scissors held in the right hand, he enters the sharp point of one of the blades under the conjunctiva at the edge of the cornea, and guiding the scissors so that it follows the circumference of the cornea, by sundry rips, divides completely the conjunctiva from the corneal border. He now seizes the cut edge of the conjunctiva, towards the inner canthus, and drawing it aside, thrusts the point of the scissors into the gaping wound and through the sub-conjunctival cellular tissue, in immediate proximity with the globe. Through this opening a strabismus hook is entered perpendicularly; its direction is then changed to a horizontal one, so as to make the end of the hook pass under the tendon of the internal rectus muscle. When drawn upon, and the tendon well exposed, it is divided near to its insertion up the sclerotic coat. The hook is now passed onwards, making the tour of the eye-ball, catching up in turn each rectus tendon, so as to facilitate its division, until all are divided by the curved scissors. Usually the order for division is as follows: First, the inner rectus is drawn out upon the hook; then the upper one, then the outer one, and finally the lower rectus is divided. The next step in the operation is to seize the sclerotic end of the internal or external rectus tendon, and forcibly rotate the eye-ball, so as to bring the optic nerve the more readily within the reach of a section. The points of a stout scissors, curved upon the flat side, are thrust into the gaping wound, and made to pass behind the eye-ball. When the optic nerve is touched, the blades are opened, the nerve caught between them and severed. From the position of the surgeon—standing at the head of the patient, with the scissors in his right hand—he will find it most convenient to divide the optic nerve by cutting from the temporal side, and therefore rotating the eye inward, if removing the right globe; whilst he would sever the optic nerve from the nasal side by rotating the left eye-ball forcibly outward. As the inelastic optic nerve held the globe in position, the cutting of this cord frees it, and permits it to spring forward when seized by the fingers of the surgeon, or lifted from its bed by using the scissors as a lever. When the globe has escaped from between the lids, the remaining attachments—the two oblique tendons—are divided close to their sclerotic insertions, and the globe is taken away perfectly clean. As soon as removed, the speculum still standing in position, a fold of wet linen is laid over the socket; a large piece of sponge is laid over this, and firmly secured in position by a piece of bandage tightly drawn around the head. The object of this sponge compress is to prevent both external hemorrhage from the cut branches of the opthalmic artery, and also to prevent any infiltration of the sub-conjunctival tissues with blood. To effect this, it should be applied immediately upon the removal of the globe from the socket. The application of the wet linen, prior to the laying on of the sponge, facilitates the removal of the latter without disturbing the bottom of the wound, which would be the case should a clot of blood connect the wound to the cellular openings of the sponge. Some pain is experienced from the binding down of the wire speculum against the lids and skin of the temple. At the expiration of from twenty to forty minutes, all tendency to hemorrhage having disappeared, the compress is removed and the speculum taken off. From this time a simple wet cloth, to be worn for one or two days, completes the dressings.

Should the socket be inspected immediately after the removal of the compress, the entire floor will be found covered by the conjunctival membrane, leaving only a small point exposed in the centre of the space, apparently corresponding to the site of the cut surface of the optic nerve. This closes over by the puckering and union of the cut edge of the conjunctiva in two or three days, thus protecting completely the socket from the danger of having bands of adhesion forming within or across the chasm. As all the muscles have been retained, and become adherent to the centre of the floor of the socket, this surface is seen to move about when movements are made in the sound eye; and when an artificial eye is applied within the perfectly-healed lids, five or six weeks after the enucleation, sufficient movements are imparted to it as will effectually restore expression to the face, and, in most cases, remove all traces of deformity.

